# Anaesthetist-provided pre-hospital advanced airway management in children: a descriptive study

**DOI:** 10.1186/s13049-015-0140-0

**Published:** 2015-08-27

**Authors:** Mona Tarpgaard, Troels Martin Hansen, Leif Rognås

**Affiliations:** Pre-hospital Critical Care Team, Aarhus University Hospital, Oluf Palmes Alle 32, Aarhus N, 8200 Denmark; Department of Pre-hospital Medical Services, Central Denmark Region, Oluf Palmes Alle 34, Aarhus N, 8200 Denmark

## Abstract

**Background:**

Pre-hospital advanced airway management has been named one of the top-five research priorities in physician-provided pre-hospital critical care. Few studies have been made on paediatric pre-hospital advanced airway management.

The aim of this study was to investigate pre-hospital endotracheal intubation success rate in children, first-pass success rates and complications related to pre-hospital advanced airway management in patients younger than 16 years of age treated by pre-hospital critical care teams in the Central Denmark Region (1.3 million inhabitants).

**Methods:**

A prospective descriptive study based on data collected from eight anaesthetist-staffed pre-hospital critical care teams between February 1st 2011 and November 1st 2012.

Primary endpoints were 1) pre-hospital endotracheal intubation success rate in children 2) pre-hospital endotracheal intubation first-pass success rate in children and 3) complications related to prehospital advanced airway management in children.

**Results:**

The pre-hospital critical care anaesthetists attempted endotracheal intubation in 25 children, 13 of which were less than 2 years old.

In one patient, a neonate (600 g birth weight), endotracheal intubation failed. The patient was managed by uneventful bag-mask ventilation. All other 24 children had their tracheas successfully intubated by the pre-hospital critical care anaesthetists resulting in a pre-hospital endotracheal intubation success rate of 96 %.

Overall first pass success-rate was 75 %. In the group of patients younger than 2 years old, first pass success-rate was 54 %.

The total rate of airway management related complications such as vomiting, aspiration, accidental intubation of the oesophagus or right main stem bronchus, hypoxia (oxygen saturation < 90 %) or bradycardia (according to age) was 20 % in children younger than 16 years of age and 38 % in children younger than 2 years of age. No deaths, cardiac arrests or severe bradycardia (heart rate <60) occurred in relation to pre-hospital advanced airway management.

**Conclusion:**

Compared with the total population of patients receiving pre-hospital advanced airway management in our system, the overall success rate following pre-hospital endotracheal intubations in children is acceptable but the first-pass success rate is low. The complication rates in the paediatric population are higher than in our pre-hospital advanced airway management patient population as a whole. This illustrates that young children may represent a substantial pre-hospital airway management challenge even for experienced pre-hospital critical care anaesthetists. This may influence future training and quality insurance initiatives in paediatric pre-hospital advanced airway management.

## Background

Tracheal intubation is still considered the golden standard for securing a definitive airway and can be lifesaving in critically ill and injured children [1].

Pre-hospital endotracheal intubation of children is rarely performed [2] and is a challenging procedure for the emergency medical care provider [3]. Some studies have shown a high rate of complications related to pre-hospital endotracheal intubation of infants and children performed by paramedics [4, 5]. In 2008 Lecky et al. concluded that in paediatric patients, the current evidence base provides no imperative to extend the practice of pre-hospital intubation in urban systems [6]. Other studies show a high pre-hospital intubation success rate [2, 7, 8]. Eich et al. [2] found an overall tracheal intubation success rate of 98,3 % (57 out of 58 children) attended by anaesthesia-trained emergency physicians. Difficult pre-hospital endotracheal intubations, defined by Cormack-Lehane score 3-4[9], was more common in infants than in older children and first-pass success rate was lower in infants. Recently, Nevin et al. [8] published a study from the UK, based on a 12 year retrospective database analysis, showing a high success rate in pre-hospital pediatric intubation performed in a physician-led trauma service. In a 12-year period, the service attended 1933 children (<16 years of age). They performed pre-hospital intubation in 315 cases, with a success rate of 99,7 %, with a single failed intubation during the study period.

An international group of experts has named pre-hospital advanced airway management (PHAAM) one of the top-five research priorities in physician-provided pre-hospital critical care [10]. A recent study in our anaesthetist-staffed pre-hospital critical care system included patients of all ages. The overall pre-hospital endotracheal intubation success rate among 636 intubation attempts was 99,7 %, first-pass success rate was 78,6 % and the overall incidence of complications related to pre-hospital advanced airway management was 7,9 %. PHAAM-related complications were defined as proposed by Sollid et al. [11] No studies have previously investigated PHAAM in children performed by the anaesthetist-staffed emergency medical services in Denmark.

The aim of this study was to describe the population of pediatric patients treated with advanced airway management by anaesthetist-staffed pre-hospital critical care teams in the Central Denmark Region [12]. The main objectives were to estimate the overall pre-hospital endotracheal intubation (PHETI) success rate, the pre-hospital endotracheal intubation first-pass success rate, and PHAAM related complications (as defined by Sollid et al. [11]) in infants and children younger than 16 years old. We furthermore wanted to compare the above mentioned results with the previously reported results in the total pre-hospital advanced airway management population in our system.

## Materials and methods

### Study design

A prospective descriptive study based on PHAAM-related data collected from eight anaesthetist-staffed pre-hospital critical care teams in the period from February 1st 2011 to November 1st 2012 in the Central Denmark Region.

### Setting

1,27 million people live in the Central Denmark Region. The region covers an approximately 13000 km^2^ mixed urban and rural area. The age distribution in percentage for children 0–14 years old is 18,1 corresponding to 229870 children younger than 16 years old. The standard EU emergency telephone number (1–1–2) covers all Denmark. There is a regional Emergency Medical Dispatch Centre and, dispatching is criteria-based [13].

The Emergency Medical Service (EMS) in the Central Denmark region has previously been described in detail [12]. In brief, the EMS consists of 64 emergency road ambulances and ten anaesthetist-staffed pre-hospital critical care teams. Nine of the teams are deployed by rapid response vehicles; the tenth team staffs a Helicopter Emergency Medical Service (HEMS). The teams covered in this study employed approximately 90 anaesthetists as part time pre-hospital critical care anaesthetists. The anaesthetists were a mixture of consultants and staff specialists and they had at least 4 ½ years experience in anaesthesia. All of them had experience with pediatric airway management from their in-hospital training and continuous work. In 2010, 18 % of the pre-hospital anaesthetists had attended the European Paediatric Life Support (EPLS) course [14]. The Advanced Peadiatric Life Support (APLS) Course is not available in Denmark.

During the study period, all pre-hospital critical care teams carried the same equipment for pediatric airway management. This included a 500 ml ventilation bag, ventilation masks size 1 to 5, endotracheal tubes size 2,5–5,0 (cuffed tubes from size 3,5) standard laryngoscope for laryngoscopy with a Macintosh blade size 0 and 1 and Miller blade size 0. As airway back-up device, the teams carried laryngeal masks (LMAs) size 1 and 2 and an AirTraq size 2. All units were equipped with a capnograph and an automated ventilator with the ability to ventilate children bigger than 10 kg. Children smaller than 10 kg were manually ventilated. The pre-hospital critical care teams carried a standardised set-up of medications including thiopental, propofol, midazolam, and s-ketamine for anaesthesia, suxametonium and rocuronium as neuro-muscular blocking agents (NMBAs) as well as different opioids for pain management. Our service had no standard operating procedure (SOP) for rapid sequence intubation (RSI) during the study period.

### Participants

The data presented in this paper are part of a larger study [12].

Inclusion criteria for this sub study: All children < 16 years of age treated with PHAAM. The definition of PHAAM was any airway management beyond opening of the airway and the use of an oro-pharyngeal airway [11].

Exclusion criteria: Interhospital transfers.

### Variables

We collected all core data proposed and defined in the consensus-based template by Sollid et al. [11].

### Descriptive variables

We registered demographic data, patient’s age and indications for performing PHAAM. The indications for performing PHAAM was, as described by Rognås et al. [12] and according to the consensus-based template by Sollid et al. [11]: 1) existing airway obstruction 2) impending airway obstruction 3) hypoxemia 4) ineffective ventilation 5) cardiac arrest 6) anaesthesia for relief of pain or distress 7) anaesthesia to combative or agitated patient 8) decreased level of consciousness 9) other.

### Exposure variables

The types of PHAAM performed by the pre-hospital critical care team were recorded as PHAAM performed without the assistance of drugs, as drug-assisted PHAAM or as RSI. We defined RSI as PHETI aided by the use of any combination of a) a sedative OR an analgesic drug AND b) an NMBA.

In this study the anaesthetists could perform PHETI by using a standard laryngoscope and for children above 10 kg it was possible to use the Airtraq^™^ laryngoscope. Other PHAAM techniques available were the use of an oropharyngeal airway or an LMA.

### Endpoints and outcome variables

Primary endpoints were 1) PHETI success rate 2) first-pass success rate and 3) complications related to PHAAM. PHETI success rate equals 1- the failed PHETI rate. Failed PHETI was defined as cases were it was not possible to establish a secure airway by endotracheal intubation. First-pass success was defined as cases were only one attempt were needed to successfully secure an airway by pre-hospital endotracheal intubation. Complications related to PHETI were defined as vomiting, aspiration of gastric content or blood to the lungs, accidental intubation of the oesophagus or right main stem bronchus, hypoxia (oxygen saturation < 90 %, bradycardia (pulse rate < 60) or dental trauma, according to the template by Sollid [11].

Secondary endpoints were the overall incidence of PHAAM-related complications linked to age, the incidence of use of airway back-up device, the reason for not doing PHETI when PHAAM was considered and a comparison of the pediatric and total PHAAM population.

### Data sources and data collection

We analysed data from the study performed by Rognås et al. [12].

### Bias

We searched the database for date of birth to collect all data of children younger than 16 years of age. (One patient first registered as a child had the age 100 years and was excluded.) As described by Rognås et al. the data were registered by the attending anaesthetists [12]. This means that they may be subject to registration bias or recall bias. The registrations were crosscheck on a daily basis against both the written pre-hospital journals and the compulsory entries made by the physicians in the patients’ hospital records, so the extent of selection bias is probably limited.

### Study size

This being a descriptive study, power calculation was waived.

### Statistical methods

Due to the nature of the study, we only used descriptive statistics.

### Ethics

The study did not involve any change from normal practice and according to Danish law, it did not need the approval of the Regional Ethics Committee. The Danish Data Protection Agency approved the study (Journal number 2013-41-1462) [12].

## Results

**Fig. 1:**
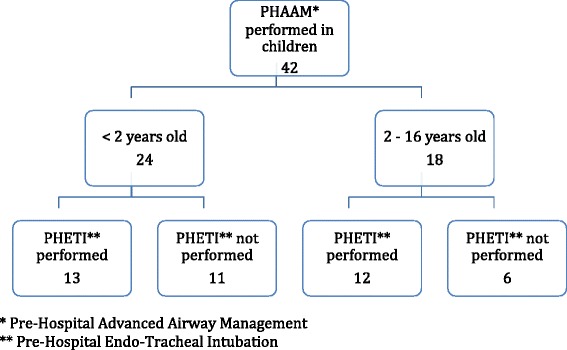
Age distribution of the included patients. *Pre-Hospital Advanced Airway Management. **Pre-Hospital Endo-Tracheal Intubation Table (vedhæftet)

Figure 1 shows the age distribution of the included patients. During the 21 months, the pre-hospital critical care teams registered data from 734 PHAAM cases. Of the 734 patients who needed PHAAM, 42 were < 16 years old. Of them, 24 patients were < 2 years old. PHETI where attempted in 25 (60 %) of these cases, 13 of them were younger than 2 years.

### Descriptive data

Table 1 shows the demographics of the paediatric population compared to the total PHAAM population.

**Table 1:** Descriptive data in the paediatric pre-hospital advanced airway management population compared with pre-hospital advanced airway management population in total

	Infant (<2 years old)	Children (<16 years of age)	Total population
Numbers	24	42	1081
Males	62,5 % (15)	52,4 % (22)	59,2 %
Pre-existing disease	45,8 % (11)	40,5 % (17)	82,5 %
Isolated TBI	4,2 % (1)	7,1 % (3)	4,2 %
Trauma in total	16,7 % (4)	23,8 % (10)	15,2 %
Cardiac arrest	33,3 % (8)	23,8 % (10)	56,4 %
Cardiac (ex cardiac arrest)	0	2,4 % (1)	3,8 %
Asthma	4,2 % (1)	2,4 % (1)	5,7 %
Other	45,8 % (11)	38,1 % (16)	16,3 %

Table 2 lists the indication for PHAAM. Tables 3 display the complications related to PHAAM and Table 4 the number of successful PHETI and first pass success rate.

**Table 2:** Indications for Pre-Hospital Advanced Airway Management in children

Indication for PHAAM^a^	All children < 16 years old (*N* = 42)	<2 years old (*n* = 24)	2 – 15 years old (*n* = 18)
Existing airway obstruction	1	1	0
Impending airway obstruction	8	5	3
Hypoxia	13	8	5
Ineffective spontaneous ventilation	9	7	2
Cardiac arrest	15	9	6
Anaesthesia to relief of pain or distress	1	1	0
Anaesthesia to combative or agitated patient	2	2	0
Decreased level of consciousness	24	13	11

^a^More than one indication may apply to each patient

**Table 3:** Complications related to Pre-Hospital Advanced Airway Management in children

	<16 years old	<2 years old	Total population
N=	42	24	1081
PHETI	25	13	735
Complications	5 (20,0 %)	4 (38,4 %)	102 (14 %)
SpO2 < 90 % after PHETI	3 (12,0 %)	2 (15,4 %)	5,2 %
Hypotension, according to age	1 (4,0 %)	0	5,2 %
Aspiration	1 (4,0 %)	0	4,6 %
Failed intubation (oesophagus)	2 (8,0 %)	2 (15,4 %)	4,3 %
Surgical trach	0	0	0,15 %

**Table 4:** Succesfull pre-Hospital endotraheal intubation and first-pass success rate

Age	<16 years old	<2 years old	Population total
N=	42	24	1081
PHAAM performed	25/42 (59,5 %)	13/24 (54,2 %)	68,0 %
PHETI attemped	25/42 (59,5 %)	13/24 (54,2 %)	63,2 %
Successful PHETI	24/25 (96,0 %)	12/13 (92,3 %)	99,7 %
First-pass success rate	18/24 (75,0 %)	7/13 (53,8 %)	77,6 %
Use of backup-device^a^	1/25 (4,0 %)	1/13 (7,6 %)	4,7 %

^a^As airway back-up device, the teams carried laryngeal masks size 1 and 2 and an AirTraq size 2.

### Primary end-points

#### Pre-hospital endotracheal intubation success rate

In one patient PHETI failed. This child was a premature newborn (birth weight 600 g) in cardiac arrest and the pre-hospital critical care anaesthetist chose to use bag-valve mask ventilation during transport to the hospital. At the hospital the child died without receiving endotracheal intubation.

Total intubation success rate was 96 % in children younger than 16 years old, and 92 % in children younger than 2 years old.

#### First-pass success rate

First-pass success-rate in all children younger than 16 years old was 75 %. In the group younger than 2 years, first pass success-rate was 54 %. In 6 of the 24 children more than one intubation attempt was needed. Five of them were younger than 2 years old.

#### Incidence of PHAAM-related complications

Complications related to PHETI were seen in 20 % of cases in patients younger than 16 years old. Among children younger than 2 years old the incidence were 38 % (4/13).

The types of complications are displayed in Table 4.

### Secondary end-points

Sixty per cent of the paediatric patients in whom PHAAM were considered had PHAAM performed. Of the children younger than 2 years old, 52 % had PHAAM done.

The Airtrach was used in 1 patient younger than 2 years old. No other airway back-up devices were used.

## Discussion

### Primary end-points

#### Pre-hospital endotracheal intubation success rate

In this study, 25 younger than 16 years old were treated with PHETI and in 96 % of the cases PHETI was successfull. Nevin et al.[8] also reports a low PHETI failure rate in their study, only a single failure in 315 cases during a 12 years period. Of the 315 children only 3 children were younger than 1 year old. In our study 13 children were younger than 2 years old. Our results compare to the results from Eich et al. [2] who reports a single PHETI failure in 58 cases. In their study 17 patients were infants (children younger than 1 year old). They found that infants, compared to older children, had a higher incidence of poor views at laryngoscopy (Cormack-Lehane grade 3 or 4) and “difficult to intubate” status. This could indicate that PHAAM are more difficult in the youngest children than in they older peers.

#### First pass success rate

Our 75 % first pass success rate in all children younger than 16 years old compares to the total PHAAM population in our service where the first pass success rate is 78 % [12]. The low first pass success rate of 54 % in the children younger than 2 years old may indicate that the smallest children constitute the most challenging PHETI-patients in the pre-hospital setting.

In addition, the often emotionally charged scene may enhance the difficulties of managing the critically sick or injured child in the pre-hospital setting. The presence of distressed relatives may provide further challenges.

Our results compares to Eich et al. [2] who reports a higher rate of difficult to intubate in infants than in older children. In 25 % (6/24), they report adverse incidents associated with endotracheal intubation in children. Adverse incidents included tracheal tube misplacement (oesophageal or endobronchial intubation) or tube dislodgement, multiple tracheal intubation attempts or failed tracheal intubation. Nevin et al. [8] reports data from a high volume, pre-hospital trauma service and over a 12 year period only 3 children under the age of 1 year had PHETI performed. According to the SOP in their system, a maximum of two initial attempts at intubation after drug administration is accepted. In case of two failed intubation attempts the practitioner is expected either to place a supraglottic device or establish a surgical airway. Neither was utilised in paediatric patients during the twelve-year period. A third alternative is bag-valve-mask ventilation while transporting the patient to hospital.

#### Incidence of PHAAM-related complications

The overall incidence of PHAAM-related complications in our service is 14,2 % as described by Rognås et al. [12]. In the paediatric population we saw a higher incidence of 20 % in children younger than 16 years of age and 38 % in children younger than 2 years of age.

Eleven of the 13 children younger than 2 year old who needed PHETI were reported to have pre-existing diseases (e.g., heart disease, pulmonary disease or diabetes). All children younger than 2 years old were described with decreased level of consciousness and in a presumed state of decompensated shock. This indicates that it may be relevant to consider whether the smallest children were sicker than the older children and the adult population when they were treated with PHETI by pre-hospital critical care teams resulting in a higher risk of PHAAM-related complications.

Post-PHAAM hypoxia (oxygen saturation < 90 % following advanced airway management) was seen in 12 % of children younger than 16 years old and 15 % of children younger than 2 years old. In the total population hypoxia was seen in 5 % of the patients. This difference may be explained by the known fact that children are more sensitive to hypoxia because of their lower functional residual capacity (the volume of air present in the lungs at the end of expiration), higher metabolism, and higher oxygen consumption.

Measurement of blood pressure can be difficult in the smallest children both because of the challenge in finding the right size of cuff and because the child may be in an agitated state. The blood pressure values must be interpreted with some caution.

Aspiration was only seen in one child older than 2 years old. This is in keeping with aspiration in general being a rare complication to RSI in children [15].

Oesophageal intubation can be fatal. It must be immediately discovered and corrected. In children with a higher incidence of poor views at laryngoscopy [16] there will be a higher risk of oesophageal intubation. In our system it is prescribed to use end-tidal CO_2_ to confirm tube placement. Two children younger than 2 years old had their oesophagus’s accidentally intubated. Both tube misplacements were immediately corrected by re-intubation.

### Secondary end-points

In the paediatric population, 60 % of the patients in whom PHAAM were considered had PHAAM performed compared to 68 % of the total population. Of the children younger than 2 years old, 52 % had PHAAM done. This could be because of a lower need for PHAAM in the smallest children. Some small children may respond surprisingly well to oxygen therapy given without manipulation of their airway.

The lower number of children who had PHAAM performed could also be because of a inclination among the anaesthetists not to perform PHETI in the youngest children. In our system the average pre-hospital anaesthetist will perform PHETI in a child every sixth year and every twelfth year in a child younger than 2 years old. This indicates that good paediatric endotracheal intubations skills cannot be acquired and maintained solely through pre-hospital practice but require on-going training and in-hospital exposure to these procedures. This is in line with a resent study from Norway by Sollid et al. [17] describe procedures performed by pre-hospital critical care anaesthetists in a helicopter emergency medical service. Among procedures expected to be performed with more than 5 years intervals, the majority are in patients younger than 12 years old. They conclude that clinical practice supported by simulation training would be the optimal method to ensure sufficient proficiency and quality in the delivery of care be anaesthetists in pre-hospital critical care medicine. In our opinion, this supports the view that paediatric pre-hospital endotracheal intubation should only be performed by trained pre-hospital critical care anaesthetist with on-going training or in-hospital exposure to these procedures.

The use of airway back-up devices in our paediatric population compares to that reported by Nevin el al.

#### Limitations

The low volume of patients in this study encumbers the results with some uncertainty. Children are included in both the paediatric population and the total population. We believe the statistical influence of the children in the total population to be low because of the low volume of children compared with adults.

We self-reported nature of the data necessitates careful interpretation. It is especially noteworthy that we do not know whether the patients who were not endotracheal intubated might have benefited from this treatment.

#### Generalizability

These data were collected from one homogenous anaesthetist-staffed EMS in one Danish region and special care should be taken before extrapolating our results to other EMS.

Our results may be of use for other physician-staffed pre-hospital critical care services with similar case load and case mix.

#### Perspectives

It would be of relevance to investigate PHETI in children in a study with a higher volume.

Our results may influence the systems for continuous paediatric pre-hospital advanced airway management training even in anaesthetist-staffed pre-hospital critical care systems.

## Conclusion

In children younger than 16 years old the overall pre-hospital endotracheal intubation success rate is high and the first-pass success rate matches that of the overall population in our service. In the age group younger than two years old the first pass success rate is considerably lower than in the rest of the population and this may indicate that the greatest airway management challenges may well be found in dealing with the smallest children.
